# Control of large amplitude limit cycle of a multi-dimensional nonlinear dynamic system of a composite cantilever beam

**DOI:** 10.1038/s41598-024-61661-8

**Published:** 2024-05-10

**Authors:** Lin Sun, Xu Dong Li, Xiaopei Liu

**Affiliations:** School of Environmental and Safety Engineering, Liaoning Petrochemical University, Fushun, 113001 China

**Keywords:** Civil engineering, Mechanical engineering, Nonlinear phenomena

## Abstract

For the first time, a control strategy based on Fuzzy Sliding Mode Control is implemented in the control of a large amplitude limit cycle of a composite cantilever beam in a multi-dimensional nonlinear form. In the dynamic model establishment of the investigated structure, the higher-order shearing effect is applied, as well as the second-order discretization. Numerical simulation demonstrates that a multi-dimensional nonlinear dynamic system of the investigated structure is demanded for accurate estimation of large amplitude limit cycle responses. Therefore, a control strategy is employed to effectively suppress such responses of the beam in multi-dimensional nonlinear form.

## Introduction

At present, composite materials are favored by research and development personnel for their characteristics of high strength and lightweight. Thus, they are widely used in high-tech industries such as aircrafts^1^, high-speed trains^2^, and ships^3^, and become the ideal materials for the fabrication of various types of cantilevered structures in engineering fields. However, such structures are prone to large-amplitude vibration triggered by external excitation, which affects the operation of these structures in engineering. Thus, controlling the large amplitude vibration of such structures is important.

Over decades, scholars have done research on various large amplitude vibrations of cantilever structures, including the limit cycle. Zhang et al.^4^ established the governing equations of a cantilever beam under combined parametric and forcing excitations assuming that the cantilever beam is an Euler–Bernoulli beam; the conditions of the limit cycle stabilization were given by Hopf bifurcation analysis, and chaotic dynamics of the cantilever beam was analyzed with a global perturbation method. Bouadjadja et al.^5^ analyzed the large deflection response of composite cantilever beams using the Euler–Bernoulli beam theory and verified it experimentally. Fu et al.^6^ investigated the thermal buckling and post-buckling of laminated composite beams based on the Timoshenko beam theory. Nguyen et al.^7^ considered Timoshenko's beam theory to derive the governing equations of composite beams considering multi-shape memory alloy layers under concentrated tip-load conditions, and primarily discussed how the temperature and layer number influence the deformation of the cantilever structure. Li et al.^8^ investigated the free vibration of laminated composite beams according to the third-order shear deformation theory and found that the length of the cantilever beam has a significant effect on the vibration mode shape. Based on a refined third-order shear deformation theory and von Kármán theory, Amabili et al.^9^ derived a nonlinear model of a self-healing composite cantilever beam through large amplitude vibration experiments. It is important to note that some of the above studies were conducted based on one-dimensional systems of cantilever beams^4^. Even in 2023, one-dimensional systems are still often used to describe periodic vibrations, bifurcations, and quasi-periodic oscillations of cantilever beams^10^.

Naturally, many scholars have applied multi-dimensional systems to study composite cantilever structures^11^. In 2013, Zhang et al.^12^ developed a two-dimensional system of a composite laminated cantilever plate under the in-plane and moment excitations and analyzed its bifurcation and chaotic motions. In 2020, Guo et al.^13^ analyzed the dynamical behaviors of a two-dimensional system of a laminated composite plate including jump, periodic, and chaotic motions. In 2022, Liu^14^ established a two-dimensional system of an axially moving composite laminated cantilever beam and discussed the influence of different axially moving rates on the tip amplitude of the axially translating structure. In the following year, Liu and Sun^15^ analyzed the chaotic responses of a composite cantilever beam in a two-dimensional form. Furthermore, in recent years, some scholars have attempted high dimensional nonlinear dynamic systems of composite cantilever structures. In 2019, Ghayesh investigated the nonlinear vibrations of functionally graded cantilevers undergoing large-amplitude oscillations using a six-mode approximation^16^; in the same year, Ghayesh also studied the large-amplitude vibrations of axially functionally graded microcantilevers in nonlinear regime using a five-mode approximation^17^; in 2022, Amabili et al. even applied a 16-mode approximation in the numerical study on the nonlinear vibration of self-healing composite cantilever beams^9^. However, there are few studies on the limit cycle of multiple-dimension nonlinear dynamic systems of composite cantilever structures.

Regarding nonlinear vibration controls in the presence of uncertainties including parametric uncertainties and external uncertainties, there have been many control strategies, and among these control strategies, Optimal Linear Feed Back Control (OLFC), State Dependent Riccati Equation Control (SDREC), Sliding Mode Control (SMC) and other nonlinear vibration control strategies are still popular research objects so far^18–21^. In 2013, both OLFC and SDREC were implemented to suppress the chaotic vibration occurred in the nonlinear dynamic system of an atomic force microscope^18^, and the sensitivity to parametric uncertainties was examined for both the control strategies; in the next year, SDREC was demonstrated to be more robust to parametric error than OLFC in nonlinear motion control of the nonlinear dynamic system of a microcantilever probe in an atomic force microscope^19^. In 2016, OLFC was proved to be not only effective in nonlinear control of an electronic circuit of a resonant MEMS mass sensor but also robust in the presence of parametric errors^20^. In addition to OLFC and SDREC, SMC was proposed by Utkin in 1992^22^, and it is widely used to control vibration together with other SMC-based strategies. In 2009, the SMC was applied to chaos control of systems with nonlinearities plus parametric uncertainties^23^; in 2018, Amin et al.^24^ proposed a robust SMC to eliminate chattering in a one-dimensional nonlinear system of the functionally graded and homogeneous nanobeams; in 2021, Youssef and Ayman utilized^25^ an integral SMC to suppress the twin-tails buffeting of a fighter aircraft. In 2006, conventional SMC was combined with fuzzy rules to alleviate the impacts of uncertainty in dynamic systems, and an innovative control method, fuzzy sliding mode control (FSMC), was proposed for Duffing system synchronization^26^. In 2016, Arun et al.^27^ implemented FSMC for Switched Reluctance Motor speed control, which has superiority over conventional PI controllers; in the next year, FSMC was validated in the nonlinear response suppression of spinning beams^28^. On the basis of FSMC, in 2007, an adaptive FSMC was developed for the control of chaotic responses discovered in Sprott's systems^29^; additionally, Ma et al.^30^ proposed an ant colony optimization-FSMC to reduce the speed chattering of pumps and compressors to enhance the cooling effect of the battery thermal management system (BTMS). It may be mentioned that the above control strategies are mostly used for one-dimensional nonlinear dynamic systems. However, to the best of the authors’ knowledge, there are few active control strategies for the control of the limit cycle of cantilever beams in multi-dimensional nonlinear dynamic form.

In this research, to control the large amplitude limit cycle of a laminated composite cantilever beam in multi-dimensional nonlinear dynamic form, an improved active control strategy is implemented. The equation of motion of the beam subject to distributed external load is firstly derived by following Hamilton’s principle, and then non-dimensionalized. The second-order Galerkin discretization is employed to obtain a two-dimensional nonlinear dynamic system. Then, the contribution of the first two vibration modes is examined, on the response of the cantilever structure. Lastly, an active control strategy available for multi-dimensional dynamic systems is employed to suppress the large amplitude limit cycle of the investigated structure.

## Model development

**Figure 1:**
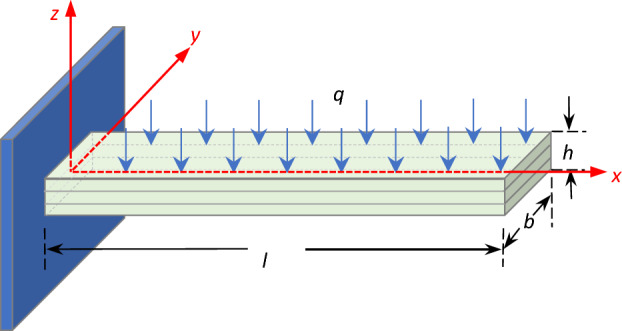
The beam structure schematic.

Figure 1 shows the schematic of the triple-layer composite cantilever beam: $l$, $b$, and $h$ denote the length, breadth, and thickness of the beam; a Cartesian coordinate is placed at the fixed boundary of the cantilever structure.

Without deformation, any point $\left(x,z\right)$ on the cantilever structure is expressed as,

$$\mathbf{r}=x\mathbf{i}+z\mathbf{k},$$

in which $\mathbf{i}$ and $\mathbf{k}$ are the unit vectors along the directions of $x$ and $z$.

Following the higher-order shear theory by Reddy, the displacement of any point after deformation is presented below,

$$\mathbf{R}=\left(x+{u}_{0}+z{\varnothing }_{x}-{z}^{3}{c}_{1}\left({\varnothing }_{x}+\frac{\partial {w}_{0}}{\partial x}\right)\right)\mathbf{i}+\left(z+{w}_{0}\right)\mathbf{k},$$

where ${c}_{1}=4/\left(3{h}^{2}\right)$, ${u}_{0}$, and ${w}_{0}$ denote the displacements along $x$ and $z$ in the middle plane $\left(z=0\right)$, and ${\varnothing }_{x}$ represents the rotation angle due to the shearing effect.

Then, the kinetic energy of the cantilever structure is obtained below,

1 $$T={\int }_{V}\frac{1}{2}\rho {\left(\frac{d\mathbf{R}}{dt}\right)}^{2}dV,$$

where $\rho$ denotes the density of the cantilever structure.

The von Kármán strain theory based on $\mathbf{r}$ is presented in the following,

$${\varepsilon }_{11}=\frac{\partial {u}_{0}}{\partial x}+\frac{1}{2}{\left(\frac{\partial {w}_{0}}{\partial x}\right)}^{2}+z\frac{\partial {\mathrm{\varnothing }}_{x}}{\partial x}-{c}_{1}{z}^{3}\left(\frac{\partial {\mathrm{\varnothing }}_{x}}{\partial x}+\frac{{\partial }^{2}{w}_{0}}{\partial {x}^{2}}\right),$$

$${\varepsilon }_{13}=\left(1-3{c}_{1}{z}^{2}\right)\left({\mathrm{\varnothing }}_{x}+\frac{\partial {w}_{0}}{\partial x}\right).$$

Therefore, the potential energy of the cantilever structure is given as follows,

2 $$U={\int }_{V}\frac{1}{2}\left({Q}_{11}{\varepsilon }_{11}{\varepsilon }_{11}+{Q}_{13}{\varepsilon }_{13}{\varepsilon }_{13}\right)dV,$$

where ${Q}_{11}$ and ${Q}_{13}$ are the elastic parameters along $x$ and $z$.

The virtual work done by both the externally applied distributed load $q$ and damping force is provided below,

3 $$W=b{\int }_{0}^{l}q{w}_{0}\left(x,t\right)dx-b{\int }_{0}^{l}c\frac{d{w}_{0}}{dt}{w}_{0}dx,$$

where $q={q}_{0}{\text{sin}}\omega t$, ${q}_{0}$, and $\omega$ are the amplitude and angular velocity of $q$, and $c$ is the damping parameter.

Following the Hamilton’s principle, it is presented as follows,

4 $${\int }_{{t}_{1}}^{{t}_{2}}\left(\delta L+\delta W\right)dt=0,$$

where $$L=T-U$$.

For an ortho-symmetric triple-layer beam, introduce Eqs. (1), (2), and (3) into Eq. (4), and then the differential dynamic equations of the cantilever structure are obtained as,

5a $${A}_{11}\frac{{\partial }^{2}{u}_{0}}{\partial {x}^{2}}+{A}_{11}\frac{\partial {w}_{0}}{\partial x}\frac{{\partial }^{2}{w}_{0}}{\partial {x}^{2}}-{I}_{0}\frac{{d}^{2}l}{d{t}^{2}}-{I}_{0}\frac{{d}^{2}{u}_{0}}{d{t}^{2}}=0,$$

5b $$\left(-{A}_{55}+6{D}_{55}{c}_{1}-9{F}_{55}{c}_{1}^{2}\right){\varnothing }_{x}+\left({D}_{11}-2{F}_{11}{c}_{1}+{H}_{11}{c}_{1}^{2}\right)\frac{{\partial }^{2}{\varnothing }_{x}}{\partial {x}^{2}}+\left(-{A}_{55}+6{D}_{55}{c}_{1}-9{F}_{55}{c}_{1}^{2}\right)\frac{\partial {w}_{0}}{\partial x}+\left(-{F}_{11}{c}_{1}+{H}_{11}{c}_{1}^{2}\right)\frac{{\partial }^{3}{w}_{0}}{\partial {x}^{3}}-{K}_{2}\frac{{d}^{2}{\varnothing }_{x}}{d{t}^{2}}+{c}_{1}{J}_{4}\frac{\partial }{\partial x}\left(\frac{{d}^{2}{w}_{0}}{d{t}^{2}}\right)=0,$$

5c $${A}_{11}\frac{\partial {w}_{0}}{\partial x}\frac{{\partial }^{2}{u}_{0}}{\partial {x}^{2}}+{A}_{11}\frac{\partial {u}_{0}}{\partial x}\frac{{\partial }^{2}{w}_{0}}{\partial {x}^{2}}+\frac{3}{2}{A}_{11}{\left(\frac{\partial {w}_{0}}{\partial x}\right)}^{2}\frac{{\partial }^{2}{w}_{0}}{\partial {x}^{2}}+{c}_{1}\left({F}_{11}-{c}_{1}{H}_{11}\right)\frac{{\partial }^{3}{\varnothing }_{x}}{\partial {x}^{3}}-{c}_{1}^{2}{H}_{11}\frac{{\partial }^{4}{w}_{0}}{\partial {x}^{4}}+\left({A}_{55}-6{c}_{1}{D}_{55}+9{c}_{1}^{2}{F}_{55}\right)\frac{\partial {\varnothing }_{x}}{\partial x}+\left({A}_{55}-6{c}_{1}{D}_{55}+9{c}_{1}^{2}{F}_{55}\right)\frac{{\partial }^{2}{w}_{0}}{{\partial }^{2}x}-{I}_{0}\frac{{d}^{2}{w}_{0}}{d{t}^{2}}+{c}_{1}^{2}{I}_{6}\frac{{\partial }^{2}}{\partial {x}^{2}}\left(\frac{{d}^{2}{w}_{0}}{d{t}^{2}}\right)-{c}_{1}{J}_{4}\frac{\partial }{\partial x}\left(\frac{{d}^{2}{\varnothing }_{x}}{d{t}^{2}}\right)=0,$$

where *A*_11_, *K*_2_, *D*_11_, *F*_11_, *H*_11_, *A*_55_, *D*_55_, *F*_55_, *I*_0_, *I*_4_, and *I*_6_ are presented in the Online Appendix A1, and,

6 $${J}_{i}={I}_{i}-{I}_{i+2}{c}_{1}, { K}_{2}=\left({I}_{2}-2{I}_{4}{c}_{1}+{I}_{6}{c}_{1}^{2}\right),$$

and $i=(0, 1, 2, \cdots ,6)$; ${\overline{Q} }_{ij}^{(1)}$, ${\overline{Q} }_{ij}^{(2)}$ and ${\overline{Q} }_{ij}^{(3)}$ denote the elastic parameters for the three layers of the cantilever structure, and ${\rho }^{(1)}$, ${\rho }^{(2)}$ and ${\rho }^{(3)}$ denote the densities for the three layers of the structure.

From Eqs. (5a) and (5b), one can derive that,

7a $$\frac{\partial {u}_{0}}{\partial x}=-\frac{1}{2}{\left(\frac{\partial {w}_{0}}{\partial x}\right)}^{2}+\frac{1}{2l}{\int }_{0}^{l}{\left(\frac{\partial {w}_{0}}{\partial x}\right)}^{2}dx+\frac{{I}_{0}}{{A}_{11}}\frac{{d}^{2}x}{d{t}^{2}}\left(x-\frac{l}{2}\right),$$

7b $${\varnothing }_{x}=-\frac{\partial {w}_{0}}{\partial x}+\frac{{F}_{11}{c}_{1}-{D}_{11}}{\left({A}_{55}-6{D}_{55}{c}_{1}+9{F}_{55}{c}_{1}^{2}\right)}\frac{{\partial }^{3}{w}_{0}}{{\partial }^{3}x}.$$

in which, comparing with the previous study^31^, both the axially moving acceleration and the axially moving velocity of the whole investigated composite cantilever structure in the present research is assumed to be zero as demonstrated below,

$${I}_{0}\frac{{d}^{2}l}{d{t}^{2}}={I}_{0}\frac{{d}^{2}x}{d{t}^{2}}=0,\frac{dl}{dt}=\frac{dx}{dt}=0.$$

Introduce Eq. (7) into Eq. (5c), it is obtained that,

8 $$-{I}_{0}\frac{{d}^{2}{w}_{0}}{d{t}^{2}}+{c}_{1}{I}_{4}\frac{{\partial }^{2}}{\partial {x}^{2}}\left(\frac{{d}^{2}{w}_{0}}{d{t}^{2}}\right)-{c}_{1}{J}_{4}\frac{{F}_{11}{c}_{1}-{D}_{11}}{\left({A}_{55}-6{D}_{55}{c}_{1}+9{F}_{55}{c}_{1}^{2}\right)}\frac{{\partial }^{4}}{{\partial }^{4}x}\left(\frac{{d}^{2}{w}_{0}}{d{t}^{2}}\right)-c\frac{d{w}_{0}}{dt}+\frac{{A}_{11}}{2{l}_{0}}\frac{{\partial }^{2}{w}_{0}}{\partial {x}^{2}}\left[{\int }_{0}^{l}{\left(\frac{\partial {w}_{0}}{\partial x}\right)}^{2}dx\right]-{D}_{11}\frac{{\partial }^{4}{w}_{0}}{{\partial }^{4}x}+{c}_{1}\left({F}_{11}-{c}_{1}{H}_{11}\right)\frac{{F}_{11}{c}_{1}-{D}_{11}}{\left({A}_{55}-6{D}_{55}{c}_{1}+9{F}_{55}{c}_{1}^{2}\right)}\frac{{\partial }^{6}{w}_{0}}{{\partial }^{6}x}+q=0.$$

## Non-dimensionalization

To validate Eq. (8)^12–15^, introduce the following non-dimension variables,

9 $$\overline{t }=\sqrt{\frac{{Q}_{11}^{(2)}I}{{I}_{0}b{l}^{4}}}t=\tau t, \overline{x }=\frac{x}{l}, \overline{l }=\frac{1}{h}l, {\overline{w} }_{0}=\frac{{w}_{0}}{h}, \frac{d{\overline{w} }_{0}}{d\overline{t} }=\frac{1}{\tau h}\frac{d{w}_{0}}{dt}, \frac{{d}^{2}{\overline{w} }_{0}}{d{\overline{t} }^{2}}=\frac{1}{{\tau }^{2}h}\frac{{d}^{2}{w}_{0}}{d{t}^{2}}, \overline{c }=c/\left(\frac{{Q}_{11}^{(2)}}{h\tau }\right),$$

where,

$$I=\underset{\Omega }{{\int }}{z}^{2}d{A}_{zy}=\frac{b{h}^{3}}{12}, {I}_{0}=\sum_{k=1}^{3}{\int }_{zk-1}^{zk}{\rho }^{(k)}dz.$$

With the non-dimension variables above substituted into Eq. (8), the non-dimensional governing equation of the beam is then obtained as follows,

10 $$-A\frac{{d}^{2}{\overline{w} }_{0}}{d{\overline{t} }^{2}}+B\frac{{\partial }^{2}}{\partial {\overline{x} }^{2}}\left(\frac{{d}^{2}{\overline{w} }_{0}}{d{\overline{t} }^{2}}\right)-C\frac{{\partial }^{4}}{\partial {\overline{x} }^{4}}\left(\frac{{d}^{2}{\overline{w} }_{0}}{d{\overline{t} }^{2}}\right)-D\frac{d{\overline{w} }_{0}}{d\overline{t} }+F\frac{{\partial }^{2}{\overline{w} }_{0}}{\partial {\overline{x} }^{2}}\left[{\int }_{0}^{1}{\left(\frac{\partial {\overline{w} }_{0}}{\partial \overline{x} }\right)}^{2}d\overline{x }\right]-G\frac{{\partial }^{4}{\overline{w} }_{0}}{\partial {\overline{x} }^{4}}+H\frac{{\partial }^{6}{\overline{w} }_{0}}{\partial {\overline{x} }^{6}}+\overline{q }=0,$$

in which, *A*, *B*, *C*, *D*, *E*, *F*, *G*, and *H* can be found in the Online Appendix A1. For convenience, ${\overline{w} }_{0}$, $\overline{x }$, $\overline{t }$, and $\overline{q }$ will be replaced with ${w}_{0}$, $x$, $t$, and $q$.

## Galerkin discretization

${w}_{0}$ Is expressed in terms of comparison functions in the following,

11 $${w}_{0}=\sum_{n=1}^{\infty }{\varphi }_{n}\left(x\right){w}_{n,1}\left(t\right).$$

Associated with the boundary conditions of the cantilever structure, $\varphi (x)$ is provided as,

$${\varphi }_{n}\left(x\right)=\left[{\text{ch}}\left({\lambda }_{n}x\right)-{\text{cos}}\left({\lambda }_{n}x\right)\right]-\frac{\left({\text{ch}}{\lambda }_{n}+{\text{cos}}{\lambda }_{n}\right)}{\left({\text{sh}}{\lambda }_{n}+{\text{sin}}{\lambda }_{n}\right)}\left[{\text{sh}}\left({\lambda }_{n}x\right)-{\text{sin}}\left({\lambda }_{n}x\right)\right],$$

where, ${\lambda }_{1}=1.875$ and ${\lambda }_{2}=4.694$ determined in the application of the second-order Galerkin discretization.

Based on the second-order Galerkin discretization, introduce Eq. (11) into Eq. (10) in the case of a specified point ${\text{P}}$ $\left(x={x}_{{\text{P}}}=0.75\right)$, it is derived that,

12 $${w}_{{\text{P}}}=\sum_{n=1}^{2}{\varphi }_{n}\left({x}_{{\text{P}}}\right){w}_{n,1}\left(t\right)=1.315382461{w}_{\mathrm{1,1}}\left(t\right)+0.27008056{w}_{\mathrm{2,1}}\left(t\right),$$

13 $$\left\{\begin{array}{c}{\dot{w}}_{\mathrm{1,1}}={w}_{\mathrm{1,2}}\\ {\dot{w}}_{\mathrm{1,2}}={T}_{11}{w}_{\mathrm{1,2}}+{T}_{12}{w}_{\mathrm{1,1}}+{T}_{13}{w}_{\mathrm{2,2}}+{T}_{14}{w}_{\mathrm{2,1}}+{T}_{15}{w}_{\mathrm{1,1}}^{3}+{T}_{16}{w}_{\mathrm{1,1}}^{2}{w}_{\mathrm{2,1}}+{T}_{17}{w}_{\mathrm{2,1}}^{2}{w}_{\mathrm{1,1}}+{T}_{18}{w}_{\mathrm{2,1}}^{3}+{T}_{19}q\\ {\dot{w}}_{\mathrm{2,1}}={w}_{\mathrm{2,2}}\\ {\dot{w}}_{\mathrm{2,2}}={T}_{21}{w}_{\mathrm{1,2}}+{T}_{22}{w}_{\mathrm{1,1}}+{T}_{23}{w}_{\mathrm{2,2}}+{T}_{24}{w}_{\mathrm{2,1}}+{T}_{25}{w}_{\mathrm{1,1}}^{3}+{T}_{26}{w}_{\mathrm{1,1}}^{2}{w}_{\mathrm{2,1}}+{T}_{27}{w}_{\mathrm{2,1}}^{2}{w}_{\mathrm{1,1}}+{T}_{28}{w}_{\mathrm{2,1}}^{3}+{T}_{29}q\end{array}\right.$$

where, ${T}_{1i}$ and ${T}_{2i}\left(i=1, 2, \cdots ,9\right)$ can be found in the Online Appendix A1.

## Large-amplitude limit cycle

A large amplitude limit cycle at ${\text{P}}$ is discovered in numerical simulation. In numerical study, the geometric coefficients of the cantilever beam are presented as follows,

14 $${l}_{0}=0.5{\text{m}}, b=0.02{\text{m}}, h=0.01{\text{m}},$$

and the external load is provided as follows,

15 $$q=3000{\text{sin}}\left(14\pi t\right){\text{Pa}}, c=0.01{\text{N}}/\left(\left({\text{m}}/{\text{s}}\right){{\text{m}}}^{2}\right),$$

and the following initial values are presented,

16 $${w}_{\mathrm{1,1}}\left(0\right)=0.3, {w}_{\mathrm{1,2}}\left(0\right)=0, {w}_{\mathrm{2,1}}\left(0\right)=0.07, {w}_{\mathrm{2,2}}\left(0\right)=0.$$

**Figure 2:**
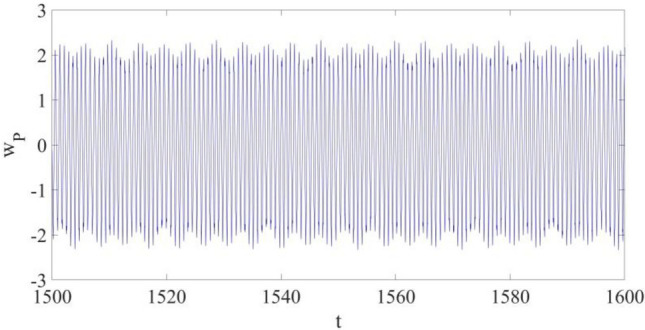
The large amplitude limit cycle at *x*_P_ = 0.75 without control.

The large amplitude limit cycle discovered based on Eqs. (12, 13) at P is presented in Fig. 2. The amplitude of the limit cycle vibration is up to around 2.3, which is nearly 2.3 times the thickness of the cantilever structure.

**Figure 3:**
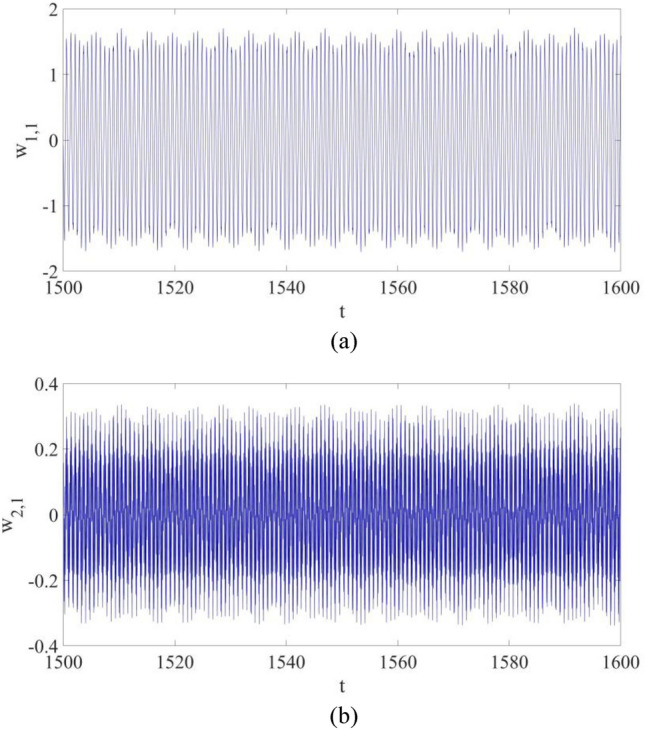
The vibrations for the first mode vibration and the second mode vibration: (**a**) ${w}_{\mathrm{1,1}}$; (**b**) ${w}_{\mathrm{2,1}}$

${w}_{\mathrm{1,1}}$ and ${w}_{\mathrm{2,1}}$ are displayed in Fig. 3. In Fig. 3a the largest amplitude of the first mode vibration is about 1.7, while in Fig. 3a the largest amplitude of the second mode vibration is about 0.34. Therefore, the influence of ${w}_{\mathrm{2,1}}$ on the actual vibration at P cannot be neglected. That is, multi-dimensional nonlinear dynamic systems of composite cantilever structures are important if a precise dynamic behavior evaluation of the structure is needed.

Then, considering the large amplitude limit cycle in Fig. 2, a control strategy available for vibration control of multi-dimensional nonlinear dynamic systems is required^15^.

## Control strategy

Based on the existing studies^28,29^, the investigated systems to be controlled can be generalized as,

17 $$\left\{\begin{array}{c}{\dot{y}}_{j}={y}_{j+1}\\ {\dot{y}}_{n}=f\left(\mathbf{Y},t\right)+d\left(\mathbf{Y},t\right)+u\\ {y}^{o}={y}_{\kappa }\end{array}\right.$$

and the corresponding reference is,

18 $$\left\{\begin{array}{c}{\dot{x}}_{j}={x}_{j+1}\\ {\dot{x}}_{n}=g\left(\mathbf{X},t\right)\\ {x}_{\kappa }^{o}={x}_{\kappa }\end{array}\right.,$$

where $1\le j\le n-1$, $\mathbf{Y}={\left[{y}_{1}{y}_{2}\cdots {y}_{n}\right]}^{T}\in {\mathbf{R}}^{n}$, $f\left(\mathbf{Y},t\right)$ gives the description of ${\dot{y}}_{n}$, $d\left(\mathbf{Y},t\right)$ is the unknown external disturbance imposed on the investigated system and is described as $\left|d\left(\mathbf{Y},t\right)\right|\le {B}_{{\text{boundary}}}\in {\mathbf{R}}^{+}$, $u\in \mathbf{R}$ denotes the control input, ${\mathbf{Y}}^{o}={\left[{x}_{1}^{o}{x}_{2}^{o}\cdots {x}_{\kappa }^{o}\right]}^{T}\left(\kappa \le j\right)$ denotes the output selected in $\mathbf{Y}$, and ${\mathbf{X}}^{o}={\left[{x}_{1}^{o}{x}_{2}^{o}\cdots {x}_{\kappa }^{o}\right]}^{T}$ is the reference vibration in response to ${\mathbf{Y}}^{o}$.

However, the control strategy shown in Eqs. (17, 18) is not applicable to a nonlinear dynamic system in multiple dimensions, such as a cantilever beam structure given in Eq. (13). The numerical results in Fig. 3, as well as the published works^12,13^, would indicate that the control strategy in Eqs. (17) and (18) cannot be applied in the vibration control of a nonlinear dynamic system in Eq. (13). Thus, an alternate control solution is developed by Liu and Sun^15^.

Following the latest study^15^, if a nonlinear dynamic equation is presented below (such as Eq. (10))

19 $$\ddot{w}=\Phi \left(w,\dot{w},t\right)$$

then, both $U$ as the control input and $\Delta F\left(w,\dot{w}\right)$ as the uncertain external perturbation can be introduced into Eq. (19) as follows,

20 $$\ddot{w}=\Phi \left(w,\dot{w},t\right)+U+\Delta F\left(w,\dot{w}\right)$$

If Eq. (11) is employed to discretize Eq. (20), a group of second-order differential equations involving $U$ and $\Delta F\left(w,\dot{w}\right)$ can be derived below,

21 $$\left\{\begin{array}{c}{\dot{w}}_{\mathrm{1,1}}={w}_{\mathrm{1,2}}\\ {\dot{w}}_{\mathrm{1,2}}={\varnothing }_{1}\left(\mathbf{W},t\right)+{u}_{1}+\Delta {f}_{1}\left(\mathbf{W},t\right)\\ {\dot{w}}_{\mathrm{2,1}}={w}_{\mathrm{2,2}}\\ {\dot{w}}_{\mathrm{2,2}}={\varnothing }_{2}\left(\mathbf{W},t\right)+{u}_{2}+\Delta {f}_{2}\left(\mathbf{W},t\right)\\ \vdots \\ {\dot{w}}_{i,1}={w}_{i,2}\\ {\dot{w}}_{i,2}={\varnothing }_{i}\left(\mathbf{W},t\right)+{u}_{i}+\Delta {f}_{i}\left(\mathbf{W},t\right)\\ \vdots \\ {\dot{w}}_{n,1}={w}_{n,2}\\ {\dot{w}}_{n,2}={\varnothing }_{n}\left(\mathbf{W},t\right)+{u}_{n}+\Delta {f}_{n}\left(\mathbf{W},t\right),\end{array}\right.$$

in which, ${\varnothing }_{i}\left(\mathbf{W},t\right)$, ${u}_{i}$, and $\Delta {f}_{i}\left(\mathbf{W},t\right)$ are the specific formulations of $\Phi \left(w,\dot{w},t\right)$, $U$, and $\Delta F\left(w,\dot{w}\right)$ obtained via the application of the Galerkin method.

Thus, $\mathbf{W}$ in Eq. (21) can be derived in the following,

22 $$\mathbf{W}={\left[{w}_{\mathrm{1,1}}{w}_{\mathrm{1,2}}{w}_{\mathrm{2,1}}{w}_{\mathrm{2,2}}\cdots {w}_{i,1}{w}_{i,2}\cdots {w}_{n,1}{w}_{n,2}\right]}^{T}.$$

Based on Eqs. (10) and (21), the nonlinear response at the selected point P is provided below,

23 $${w}_{{\text{P}}}=\sum_{n=1}^{\infty }{\varnothing }_{n}\left({x}_{{\text{P}}}\right){w}_{n,1}\left(t\right),$$

in which ${x}_{{\text{P}}}$ represents the position of P.

A reference is given below,

24 $${w}_{r}=\Psi \left(t\right).$$

$U$ is presented as,

25 $$U={U}_{eq}-{U}_{r},$$

where ${U}_{eq}$ and ${U}_{r}$ are given as follows,

26 $${U}_{eq}=-\left(\left({\dot{w}}_{{\text{P}}}-\dot{\Psi }\right)+\kappa \left({w}_{{\text{P}}}-\Psi \right)\right), {U}_{r}={k}_{fs}{U}_{fs}.$$

In Eq. (26), $\kappa$ denotes the control coefficients governing the sliding surface, ${k}_{fs}$ is defined as $\left|\Delta F\left(w,\dot{w}\right)\right|<{k}_{fs}\in {\mathbf{R}}^{+}$, and ${U}_{fs}$ follows the fuzzy rule in Table 1^26^.

**Table 1:** The fuzzy rule of $U_{fs}$.

$U_{fs}$		*U*_*eq*_						
		PB	PM	PS	ZE	NS	NM	NB
$\frac{{dU_{eq} }}{dt}$	PB	NB	NB	NB	NB	NM	NS	ZE
	PM	NB	NB	NB	NM	NS	ZE	PS
	PS	NB	NB	NM	NS	ZE	PS	PM
	ZE	NB	NM	NS	ZE	PS	PM	PB
	NS	NM	NS	ZE	PS	PM	PB	PB
	NM	NS	ZE	PS	PM	PB	PB	PB
	NB	ZE	PS	PM	PB	PB	PB	PB

**Figure 4:**
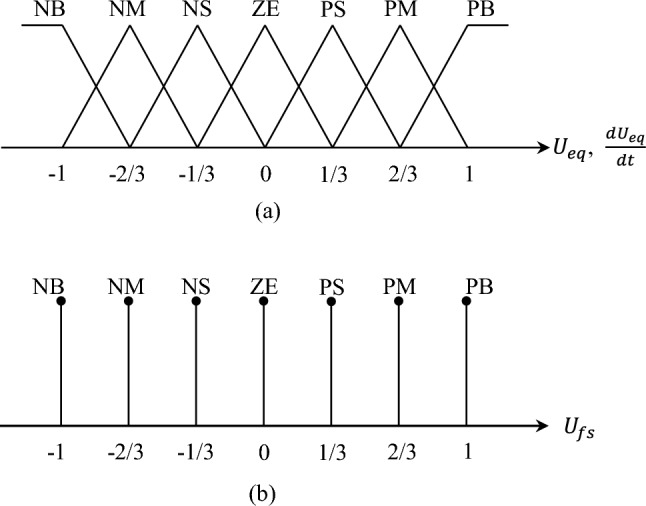
Memberships for control variables: (**a**) ${U}_{eq}$, $\frac{d{U}_{eq}}{dt}$; (**b**) ${U}_{fs}$.

Besides Table 1, the memberships for, ${U}_{eq}$, $\frac{d{U}_{eq}}{dt}$, and ${U}_{fs}$ are shown in Fig. 4a,b^27,28^.

With the control strategy in Eqs. (21–26), the active vibration control of the large amplitude limit cycle discovered based on the governing equation in Eq. (10) is ready. Based on $U$ and $\Delta F\left(w,\dot{w}\right)$ in Eq. (20), the governing equation in Eq. (10) gives the following,

27 $$-A\frac{{d}^{2}{w}_{0}}{d{t}^{2}}+B\frac{{\partial }^{2}}{\partial {x}^{2}}\left(\frac{{d}^{2}{w}_{0}}{d{t}^{2}}\right)-C\frac{{\partial }^{4}}{\partial {x}^{4}}\left(\frac{{d}^{2}{w}_{0}}{d{t}^{2}}\right)-D\frac{d{w}_{0}}{dt}+F\frac{{\partial }^{2}{w}_{0}}{\partial {x}^{2}}\left[{\int }_{0}^{1}{\left(\frac{\partial {w}_{0}}{\partial x}\right)}^{2}dx\right]-G\frac{{\partial }^{4}{w}_{0}}{\partial {x}^{4}}+H\frac{{\partial }^{6}{w}_{0}}{\partial {x}^{6}}+q-U-\Delta F\left({w}_{0},{\dot{w}}_{0}\right)=0.$$

Applying the second-order Galerkin discretization, it can be obtained from Eq. (27) as follows,

28 $$\left\{\begin{array}{c}{\dot{w}}_{\mathrm{1,1}}={w}_{\mathrm{1,2}}\\ {\dot{w}}_{\mathrm{1,2}}={T}_{11}{w}_{\mathrm{1,2}}+{T}_{12}{w}_{\mathrm{1,1}}+{T}_{13}{w}_{\mathrm{2,2}}+{T}_{14}{w}_{\mathrm{2,1}}+{T}_{15}{w}_{\mathrm{1,1}}^{3}+{T}_{16}{w}_{\mathrm{1,1}}^{2}{w}_{\mathrm{2,1}}+{T}_{17}{w}_{\mathrm{2,1}}^{2}{w}_{\mathrm{1,1}}\\ +{T}_{18}{w}_{\mathrm{2,1}}^{3}+{T}_{19}q+{u}_{1}+\Delta {f}_{1}\left(\mathbf{W},t\right)\\ {\dot{w}}_{\mathrm{2,1}}={w}_{\mathrm{2,2}}\\ {\dot{w}}_{\mathrm{2,2}}={T}_{21}{w}_{\mathrm{1,2}}+{T}_{22}{w}_{\mathrm{1,1}}+{T}_{23}{w}_{\mathrm{2,2}}+{T}_{24}{w}_{\mathrm{2,1}}+{T}_{25}{w}_{\mathrm{1,1}}^{3}+{T}_{26}{w}_{\mathrm{1,1}}^{2}{w}_{\mathrm{2,1}}+{T}_{27}{w}_{\mathrm{2,1}}^{2}{w}_{\mathrm{1,1}}\\ +{T}_{28}{w}_{\mathrm{2,1}}^{3}+{T}_{29}q+{u}_{2}+\Delta {f}_{2}\left(\mathbf{W},t\right)\end{array}\right.$$

where, ${u}_{1}$ and ${u}_{2}$ are derived with the second-order Galerkin discretization below,

$${u}_{1}=0.7849249756U, {u}_{2}=0.4319801434U.$$

## Vibration control

In this section, the control of the limit cycle at the selected point is applied via the strategy demonstrated in the previous section. Firstly, the control strategy developed in the previous section is implemented with only external disturbance considered; then, combined with the externally imposed disturbance, the control strategy is applied in the presence of parametric error to examine the influence of parametric errors on the performance of the used control design.

**(i) Subject to external disturbance**

**Figure 5:**
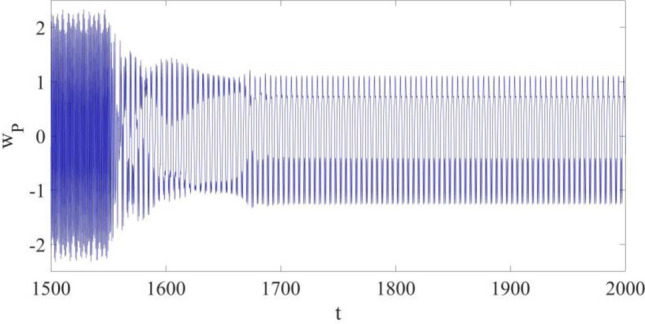
The response of the cantilever structure at ${x}_{{\text{P}}}=0.75$ with control.

**Figure 6:**
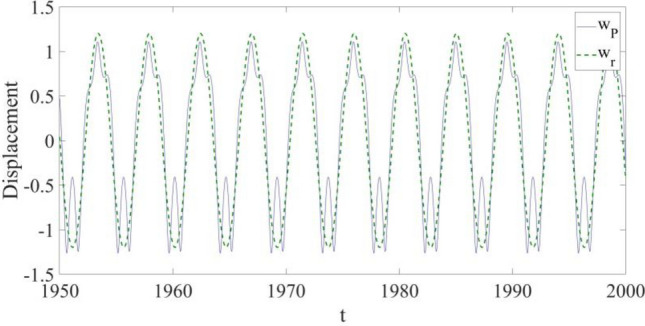
The displacement comparison between the response at ${x}_{{\text{P}}}=0.75$ and the response of the reference.

**Figure 7:**
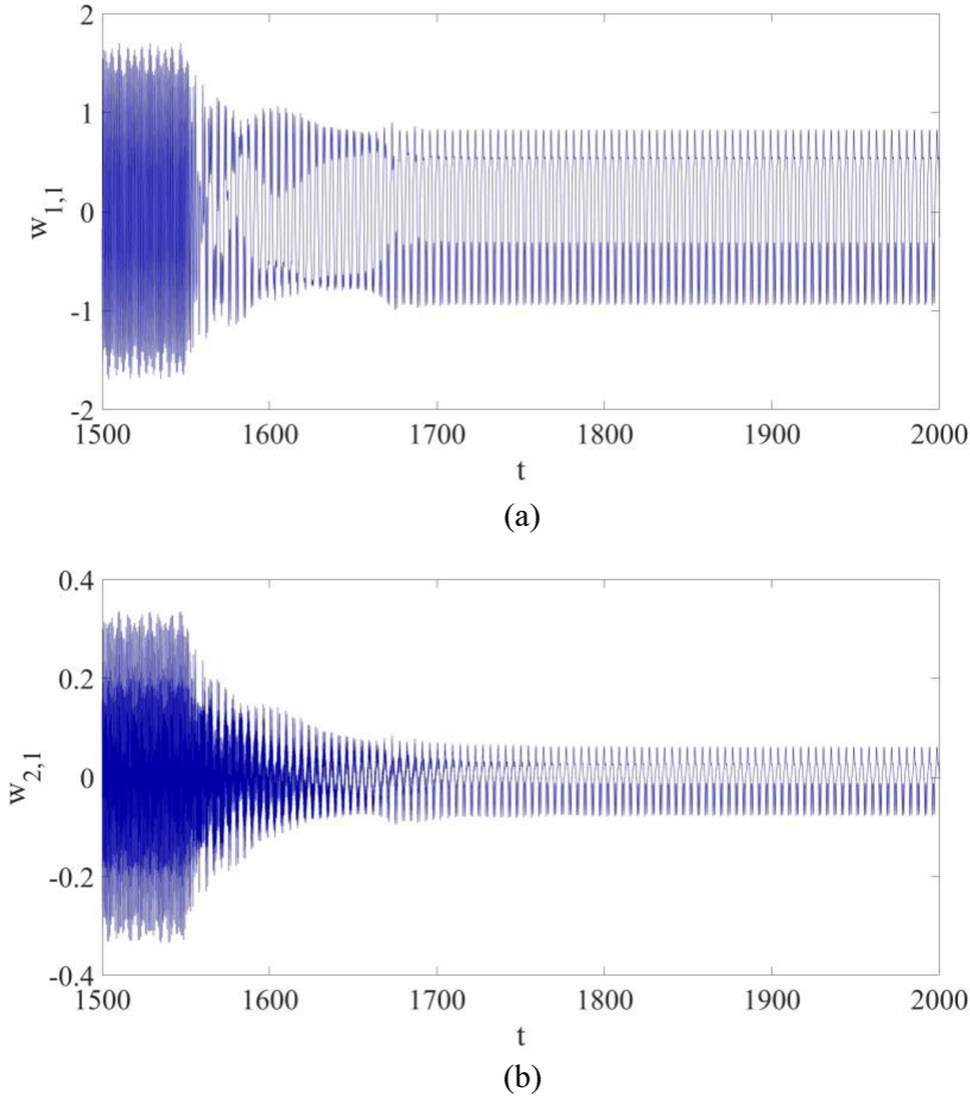
The vibrations for the first mode vibration and the second mode vibration with control: (**a**) ${w}_{\mathrm{1,1}}$; (**b**) ${w}_{\mathrm{2,1}}$.

The vibration control is conducted at *t* = 1550 as shown in Figs. 5, 6 and 7, and the control coefficients are provided as follows,

29 $${w}_{r}=1.2{\text{sin}}\left(1.3903t\right), \kappa =0.1, {k}_{fs}=0.1,\Delta F\left(w,\dot{w}\right)=0.01{\text{sin}}\left({w}_{{\text{P}}}\right).$$

In Fig. 5, the maximum amplitude of the limit cycle at P is reduced by 52.174% from about 2.3 to 1.1, and the actual vibration at the specified point is suppressed and controlled with ${w}_{r}$; the synchronization process takes about 125 non-dimensional time units.

In Fig. 6, a transverse displacement comparison is shown to examine the effectiveness of the vibration control in detail, and it is learned: the vibration at the selected point P is close to that of ${w}_{r}$, despite some small discrepancies existing in the areas where the transverse displacement of the structure vibration is at its largest values.

Figure 7 shows the vibrations of the first mode and the second mode at the selected point P: both ${w}_{\mathrm{1,1}}$ and ${w}_{\mathrm{2,1}}$ eventually become periodic vibrations with control, and their largest amplitudes both decrease significantly.

**Figure 8:**
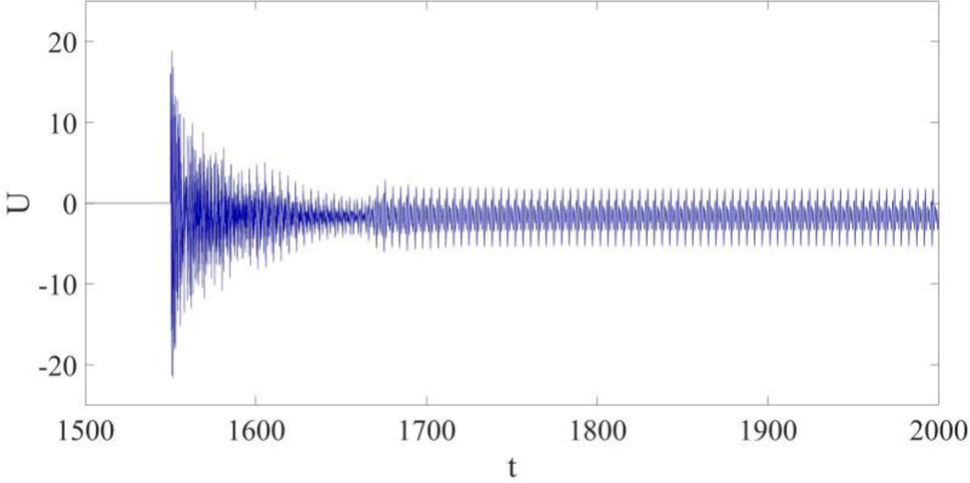
Control input.

The control input is shown in Fig. 8. Firstly, $U$ increases to the largest value within the whole control progress the moment the control is implemented, and its largest value is about 21. During the vibration suppression progress, which begins at the time *t* = 1550, $U$ gradually decreases, and finally becomes stabilized when the vibration at P is synchronized with ${w}_{r}$. It is found out: after the large amplitude limit cycle is successfully suppressed, only a relatively low control cost is required to keep vibration synchronization.

**(ii) Subject to external disturbance and parametric errors**

The vibration control is conducted in the presence of both external disturbance and parametric errors. While the previous control configuration remains the same, the parameters ${T}_{1i}$ and ${T}_{2i}$ in Eq. (13) are considered to have a random error of $\pm 8\%$ as follows,

30 $${T}_{1i}^{*}=\left(1+0.08{r}_{1i}\left(t\right)\right){T}_{1i},{T}_{2i}^{*}=\left(1+0.08{r}_{2i}\left(t\right)\right){T}_{2i}$$

in which, $i = 1, 2, \ldots ,9$, and ${r}_{1i}$ and ${r}_{2i}$ are normally distributed random functions based on the previous study by Shirazi et al.^32^.

**Figure 9:**
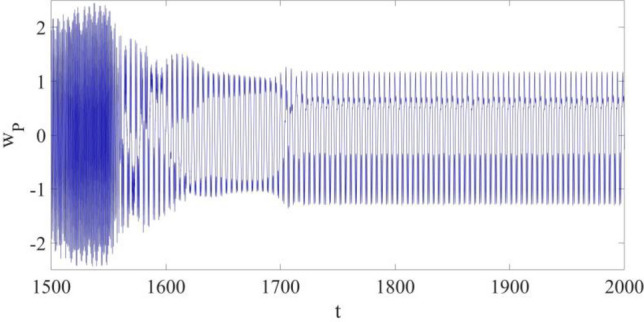
The response of the cantilever structure at ${x}_{{\text{P}}}=0.75$ with control.

With ${T}_{1i}^{*}$ and ${T}_{2i}^{*}$ substituted into Eq. (13), the actual vibration at P is shown in Fig. 9. It can be seen that the vibration at P can still be suppressed and controlled, while the synchronization process takes longer time. Specifically, the synchronization process takes about 170 non-dimensional time units, which is about 1/3 longer than that of the previous case (125 non-dimensional time units).

**Figure 10:**
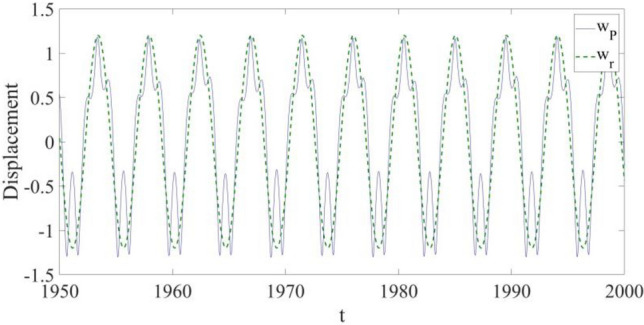
The displacement comparison between the response at ${x}_{{\text{P}}}=0.75$ and the response of the reference.

The transverse displacement comparison in Fig. 10 shows the effectiveness of the vibration control in the presence of both external disturbance and parametric errors. It can be seen that the vibration at the selected point P is still close to that of ${w}_{r}$, except some small discrepancies in the areas where the transverse displacement of the structure reaches its largest values. Furthermore, the suppressed vibration is also similar to the suppressed vibration of the previous case, and therefore the robustness of the used control design is demonstrated in the presence of both external disturbance and parametric errors.

**Figure 11:**
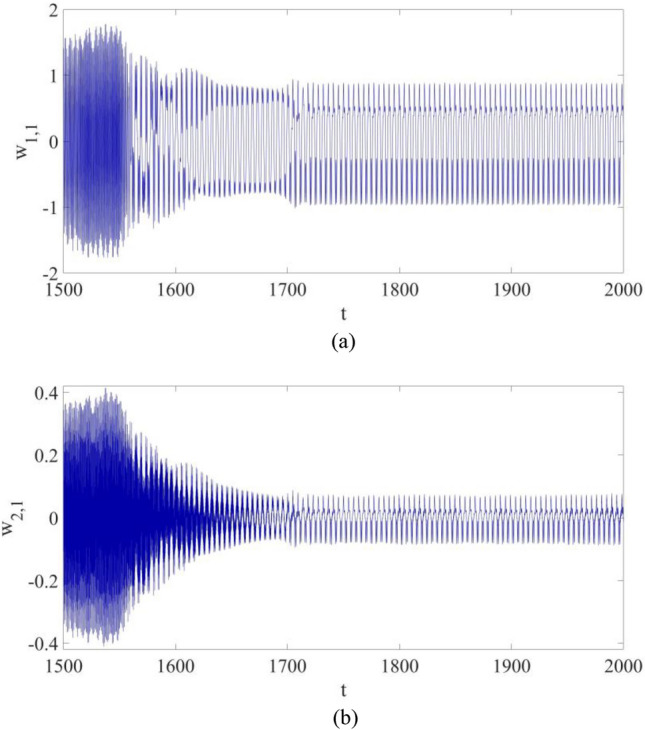
The vibrations for the first mode vibration and the second mode vibration with control: (**a**) ${w}_{\mathrm{1,1}}$; (**b**) ${w}_{\mathrm{2,1}}$.

Figure 11 shows the vibrations of ${w}_{\mathrm{1,1}}$ and ${w}_{\mathrm{2,1}}$. It can be found out that both of them eventually become suppressed with control applied, while more synchronization time is demanded probably due to the introduction of parametric errors.

**Figure 12:**
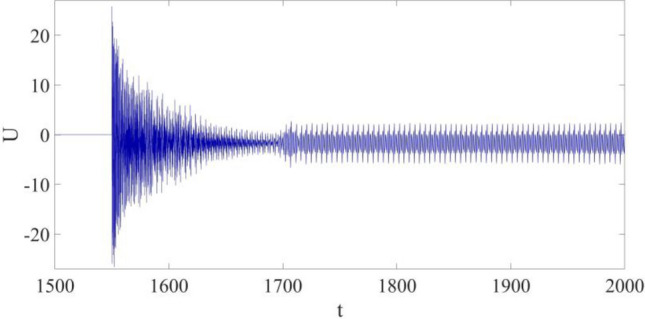
Control input.

The control input is shown in Fig. 12. Similarly, $U$ still quickly increases to the largest value at the beginning of the control application, and then decreases to a relatively smaller value once the vibration is controlled. However, in this case, the largest control input required within the whole control process is about 27, which is about 1/3 larger than the value 21 as mentioned in the previous case. In addition, the control input demanded to maintain the synchronization is also higher than that of the previous case. That is, the control cost is higher probably due to the introduction of parametric errors.

## Conclusions

For the first time, the control of the large amplitude limit cycle of a laminated composite cantilever beam structure is performed. In simulation, a large amplitude limit cycle of the cantilever beam is discovered, and it is demonstrated that a multiple-dimension nonlinear dynamic system is necessary for accurate vibration estimation of the investigated cantilever structure. Hence, the latest proposed control strategy is implemented, which is available for such multiple-dimension nonlinear systems. The numerical simulation proves both the validity and efficiency of the vibration control of large amplitude limit cycle vibrations in multiple-dimension nonlinear systems, while the robustness of the used control design is demonstrated against small-range of parametric errors at the cost of longer synchronization time and higher control input.

## Future development

Based on the demonstrated applicability of the proposed control strategy in the two-dimensional nonlinear dynamic model of composite cantilever beams in the present study, the control strategy should be further examined for high-dimensional models, such as five-dimensional models^17^ and even sixteen-dimensional models^9^. In addition to the control of the large amplitude limit cycle of a laminated composite cantilever beam structure, other responses of the engineering structures involving the studied structure such as buckling and chaotic vibrations should be considered in active vibration controls to ensure the operation and structure health of those engineering structures, and in the future control applications the control strategy employed in the current research should also be adapted and carefully examined if necessary modification is required in the case of buckling or chaotic vibration controls. Also, considering the fact that small-range parametric error results in significant increase in synchronization time and control cost, the vibration control of the studied laminated composite cantilever beam structure should also be performed to further analyze the influence of different kinds of parametric errors on the performance of the used control design, and other established control strategies including OLFC and SDREC may be compared in future study.

## Supplementary Information

Supplementary Information.

## Data Availability

Data is provided within the manuscript, and the detailed datasets used and analyzed in the current study are available from the corresponding author upon request.
