# Supplementary material for: Control of large amplitude limit cycle of a multi-dimensional nonlinear dynamic system of a composite cantilever beam
Source: Sci Rep. 2024 May 10;14:10771. doi: 10.1038/s41598-024-61661-8 (PMC11649825; doi:10.1038/s41598-024-61661-8)
Supplement: Supplementary file 1 — Supplementary Information. [file 41598_2024_61661_MOESM1_ESM.docx]

**Appendix**

$\left( A_{11},D_{11},F_{11},H_{11} \right)=\sum_{k=1}^{3} \int_{z_{k}}^{z_{k+1}} Q_{11}^{\left( k \right)}\left( 1,z^{2},z^{4},z^{6} \right)dz$,

$\left( A_{55},D_{55},F_{55} \right)=\sum_{k=1}^{3} \int_{z_{k}}^{z_{k+1}} Q_{13}^{\left( k \right)}\left( 1,z^{2},z^{4} \right)dz$,

$\left( I_{0},I_{2},I_{4},I_{6}, \right)=\sum_{k=1}^{3} \int_{z_{k}}^{z_{k+1}} \rho^{\left( k \right)}\left( 1,z^{2},z^{4},z^{6} \right)dz$;

$A=\frac{I_{0}}{\rho h}$, $B=\frac{1}{l^{2}}\frac{c_{1}I_{4}}{\rho h}$, $C=\frac{1}{l^{4}}\frac{c_{1}J_{4}}{\rho h}\left( \frac{F_{11}c_{1}-D_{11}}{A_{55}-6D_{55}c_{1}+9F_{55}c_{1}^{2}} \right)$, $D=\frac{c}{\rho h\tau}$, $F=\frac{hA_{11}}{2\rho\tau^{2}l^{4}}$, $G=\frac{D_{11}}{\rho h\tau^{2}l^{4}}$,

$H=\frac{c_{1}\left( F_{11}-c_{1}H_{11} \right)}{\rho h\tau^{2}l^{6}}\left( \frac{F_{11}c_{1}-D_{11}}{A_{55}-6D_{55}c_{1}+9F_{55}c_{1}^{2}} \right)$;

$T_{11}=U_{1}S_{1b}+U_{2}S_{2b}$, $T_{12}=U_{1}S_{1c}+U_{2}S_{2c}$, $T_{13}=U_{1}S_{1e}+U_{2}S_{2e}$,

$T_{14}=U_{1}S_{1f}+U_{2}S_{2f}$, $T_{15}=U_{1}S_{1g}+U_{2}S_{2g}$, $T_{16}=U_{1}S_{1h}+U_{2}S_{2h}$,

$T_{17}=U_{1}S_{1i}+U_{2}S_{2i}$, $T_{18}=U_{1}S_{1j}+U_{2}S_{2j}$, $T_{19}=U_{1}S_{1k}+U_{2}S_{2k}$;

$T_{21}=U_{3}S_{1b}+U_{4}S_{2b}$, $T_{22}=U_{3}S_{1c}+U_{4}S_{2c}$, $T_{23}=U_{3}S_{1e}+U_{4}S_{2e}$,

$T_{24}=U_{3}S_{1f}+U_{4}S_{2f}$, $T_{25}=U_{3}S_{1g}+U_{4}S_{2g}$, $T_{26}=U_{3}S_{1h}+U_{4}S_{2h}$,

$T_{27}=U_{3}S_{1i}+U_{4}S_{2i}$, $T_{28}=U_{3}S_{1j}+U_{4}S_{2j}$, $T_{29}=U_{3}S_{1k}+U_{4}S_{2k}$;

$U_{1}=-\frac{S_{2d}}{S_{1a}S_{2d}-S_{1d}S_{2a}}$, $U_{2}=\frac{S_{1d}}{S_{1a}S_{2d}-S_{1d}S_{2a}}$, $U_{3}=\frac{S_{2a}}{S_{1a}S_{2d}-S_{1d}S_{2a}}$,

$U_{4}=-\frac{S_{1a}}{S_{1a}S_{2d}-S_{1d}S_{2a}}$;

$S_{1a}=-1.000000000A+0.8581959666B-12.35961914C$,

$S_{1b}=-1.000000000D$,

$S_{1c}=+2.005226046E-12.35961914G+10.60697530H$,

$S_{1d}=-11.74232364B$,

$S_{1e}=0.0000000000$,

$S_{1f}=-6.312477510E-5700.676306H$,

$S_{1g}=+3.994625840F$,

$S_{1h}=-67.36709586F$,

$S_{1i}=+201.8072370F$,

$S_{1j}=-381.7008418F$,

$S_{1k}=+0.7829242108$;

$S_{2a}=+1.873752475B$,

$S_{2b}=0.0000000000$,

$S_{2c}=+0.5091766256E+23.15886696H$,

$S_{2d}=-1.000000000A-13.29402727B-485.4811154C$,

$S_{2e}=-1.000000000D$,

$S_{2f}=+2.018651170E-485.4811154G-6453.999206H$,

$S_{2g}=+8.721714324F$,

$S_{2h}=-89.63073490F$,

$S_{2i}=+257.8009342F$,

$S_{2j}=-432.1411636F$,

$S_{2k}=+0.4339831449$.
